# Computational identification of immune-related lncRNA signature for predicting the prognosis and immune landscape of human glioblastoma multiforme

**DOI:** 10.3389/fimmu.2022.932938

**Published:** 2022-08-12

**Authors:** Dongjie Shi, Wenjie Zhong, Dan Liu, Xiaochuan Sun, Shilei Hao, Yaying Yang, Lei Ao, Junjie Zhou, Yongzhi Xia, Yudong Zhou, Hua Yu, Haijian Xia

**Affiliations:** ^1^ Department of Neurosurgery, The First Affiliated Hospital of Chongqing Medical University, Chongqing, China; ^2^ Department of Pharmacy, Chongqing General Hospital, University of Chinese Academy of Sciences, Chongqing, China; ^3^ Key Laboratory of Biorheological Science and Technology, Ministry of Education, College of Bioengineering, Chongqing University, Chongqing, China; ^4^ Department of Pathology, Molecular Medicine and Tumor Center, Chongqing Medical University, Chongqing, China; ^5^ Department of Neurosurgery, Children’s Hospital of Chongqing Medical University, Chongqing, China

**Keywords:** glioblastoma multiforme, immune-related long noncoding RNAs, prognosis, immune checkpoint inhibitors, effectiveness

## Abstract

Emerging evidence shows immune-related long noncoding RNAs (ir-lncRNAs) perform critical roles in tumor progression and prognosis assessment. However, the identification of ir-lncRNAs and their clinical significance in human glioblastoma multiforme (GBM) remain largely unexplored. Here, a designed computational frame based on immune score was used to identify differentially expressed ir-lncRNAs (DEir-lncRNAs) from The Cancer Genome Atlas (TCGA) GBM program. The immune-related lncRNA signature (IRLncSig) composed of prognosis-related DEir-lncRNAs selected by Cox regression analysis and its clinical predictive values were verified, which was further validated by another dataset from the Gene Expression Omnibus database (GEO). Subsequently, the association between IRLncSig and immune cell infiltration, immune checkpoint inhibitor (ICI) biomarkers, O6-methylguanine-DNA methyltransferase (MGMT) gene expression, and biological function were also analyzed. After calculation, five prognosis-related ir-lncRNAs were included in the establishment of IRLncSig. The risk assessment based on IRLncSig indicated that the high-IRLncSig-score group was significantly associated with poor prognosis (*p* < 0.001), significant aggregation of macrophages (*p* < 0.05), higher ICI biomarker expression, and MGMT gene expression (*p* < 0.05). Signature-related lncRNAs may be involved in immune activities in the tumorigenesis and progression of GBM. In summary, the novel IRLncSig shows a promising clinical value in predicting the prognosis and immune landscape of GBM.

## Introduction

Glioblastoma multiforme (GBM) is one of the most aggressive and deadly malignant solid tumors. It has been reported that the 5-year survival rate of 12,120 newly diagnosed glioblastoma patients in the United States is only 5% (1). Even with great efforts, conventional treatment strategies poorly improve the prognosis of GBM patients. With the combination of existing treatment strategies, including maximal-safe surgical resection, adjuvant radiation therapy, and adjuvant chemotherapy, the patient’s median survival time is only 15 months (2). Thus, more effective treatment strategies are urgently needed to improve the prognosis of GBM patients.

In recent years, immunotherapy, which mainly depends on activating innate immune cells in the tumor microenvironment (TME), has been widely recognized as an effective treatment strategy for tumors. An emerging study demonstrated that innate immune cells change the TME and gradually weaken human immune surveillance with cytokines during tumor development (3). With antiprogrammed cell death protein-1 (PD-1)/programmed death ligand-1 (PD-L1) used as the first clinical immune checkpoint inhibitors (ICIs), many different types of immune checkpoint inhibitors have been gradually used for cancer treatment. According to reports, PD-1/PD-L1 inhibitors can induce long-term remission in some breast cancer subtype patients and induce long-term immune responses to tumors (4). PD-1/PD-L1 inhibitors can significantly improve overall and progression-free survival for previously treated patients with advanced nonsmall-cell lung cancer (NSCLC) (5). A preclinical trial demonstrated that the combination of a PD-1 inhibitor and radiotherapy significantly increased the survival time of mice compared to other single treatments for orthotopic brain tumors (6). In the subsequent phase III CheckMate 143 trial of nivolumab (NCT02017717), no significant improvement was observed in the median survival and 12-month survival rates of recurrent GBM treated with a PD-1 inhibitor compared with the vascular endothelial growth factor A (VEGF-A) inhibitor bevacizumab (7). Regarding the safety of ICIs, a study showed that combination therapy of ICIs (PD-1 inhibitor and cytotoxic T-lymphocyte-associated antigen-4 (CTLA4) inhibitor) caused serious side effects and lead to the termination of the trial (8). However, another clinical trial suggested that pembrolizumab (a PD-1 inhibitor) combined with radiotherapy and bevacizumab in patients with recurrent high-grade glioma showed better survival (9). Therefore, creating meaningful classifiers that can effectively evaluate ICI effectiveness for GBM patients and stratify patients to achieve precision medical care is a challenge.

The human genome is widely transcribed, but only approximately 2% of expressed transcripts can encode proteins, and the remaining transcripts longer than 200 t are defined as long noncoding RNAs (lncRNAs). Further studies of lncRNAs demonstrated the pathological process of some incurable diseases, especially the occurrence of cancer. Emerging studies have shown that lncRNAs are closely related to tumor immune activities. The lncRNA small nucleolar RNA host gene 1 (SNHG1) can affect the immune escape of breast cancer by regulating the differentiation of Treg cells (10). By stabilizing the PD-1 protein and degrading the GATA binding protein 3 (GATA3), the lncRNA GATA binding protein 3 antisense RNA 1 (GATA3-AS1) promotes the progression and immune evasion of triple-negative breast cancer (11). The expression of some immune-related lncRNAs (ir-lncRNAs) was found to be closely related to GBM. Immune-related lncRNA (DiGeorge syndrome critical region gene 5 (DGCR5) expression is downregulated in glioma, and high expression independently predicts better prognosis in glioma patients (12). A higher expression of lncRNA MIR155 Host Gene (MIR155HG) was associated with worse overall survival (OS) in GBM (13). Some previous studies reported that signatures established by ir-lncRNAs can effectively predict the prognosis and immunotherapeutic responses of different tumors, but no such study has been performed in GBM (14–17). Therefore, we first used ir-lncRNAs to construct an immune-related lncRNA signature (IRLncSig) as a classifier to predict the prognosis of GBM patients and stratify patients to obtain more effective individualized treatment.

In this study, the GBM patients were defined as immune-score-high (IH) and immune-score-low (IL) groups based on the immune score in The Cancer Genome Atlas (TCGA) GBM cohort. Based on the expression data, the differentially expressed ir-lncRNAs (DEir-lncRNAs) were screened to construct IRLncSig. The prognostic predictive value of the IRLncSig was then estimated among patients with GBM. The correlations between IRLncSig and immune cell infiltration, ICI biomarkers, and O6-methylguanine-DNA methyltransferase (MGMT) gene expression were also analyzed.

## Methods

### Data acquisition

Transcriptome profiling (RNA-seq, data type: counts) of GBM was obtained from TCGA (https://tcga-data.nci.nih.gov/tcga/). After checking the sample processing information, most samples that met the requirements were enrolled in the research. All included transcriptome profiles were annotated by gene transfer format (GTF) files (downloaded from Ensembl (http://asia.ensembl.org)) to distinguish messenger RNAs (mRNAs) and lncRNAs. Immune-related gene (ir-gene) profiles were obtained from the ImmPort database (http://www.immport.org). Estimation of Stromal and Immune cells in Malignant Tumor tissues using Expression data (ESTIMATE) profiles were downloaded from the ESTIMATE database (https://bioinformatics.mdanderson.org/estimate/). The independent GBM validation set GSE53733 (18) with transcriptome data and survival information was obtained from the Gene Expression Omnibus database (GEO). All enrolled samples’ clinical information was retrieved from the GBM project of TCGA. The obtained data were screened, and unnecessary information was deleted. All enrolled samples were ensured that there were no missing values in the clinical survival status and overall survival time.

### Establishment and evaluation of an immune-related lncRNA signature

Coexpression analyses were used between ir-genes and transcriptome data to identify ir-lncRNAs (*r* > 0.6 and *p*-value <0.001). The median immune score from ESTIMATE profiles was used to divide all samples into IL and IH groups. The R package DESeq2 was used to identify the DEir-lncRNAs between the two groups. A log fold change (logFC) > 1.0 and false discovery rate (FDR) <0.05 were regarded as the cutoff values.

Univariate and multivariate Cox proportional hazard regression analyses were used to screen out ir-lncRNAs whose expression levels were significantly associated with patient overall survival. An IRLncSig was constructed by the coefficients from the multivariate regression analysis and the count of selected prognostic-related ir-lncRNA expression. The IRLncSig score of each sample was calculated by expression data. The median of the IRLncSig score was used to assign the GBM patients to a high-IRLncSig-score group and a low-IRLncSig-score group. The Kaplan–Meier analysis was used to evaluate the survival differences of patients in different groups, and the results were visualized. Next, the IRLncSig was further validated using another dataset from the GEO database (GSE53733). The survival outcome, IRLncSig score, and lncRNA expression patterns were also visualized by R tools (Version: 4.0.3). Univariate and multivariate Cox regression analyses between the IRLncSig score and clinical feature characteristics were then performed to evaluate whether IRLncSig can be used as an independent clinical prognostic predictor. A time-dependent receiver operating characteristic (ROC) curve was used to verify the clinical performance of IRLncSig. All R packages used in this process included survival, survivalROC, pheatmap, and survminer.

### Biological function of IRLncSig

Marker-gene-based approaches (M) [MCP-counter (19) and xCell (20, 21)] and deconvolution-based approaches (D) [CIBERSORT (22, 23), CIBERSORT-ABS (24), EPIC (25), quanTIseq (26, 27), and TIMER (28, 29)] were used to perform immune infiltration analysis (30). CIBERSORT was used to conduct intrasample comparisons between cell types and others for intersample comparisons of the same cell type. First, the Spearman’s correlation analysis was used to analyze the potential relationship between the IRLncSig score and TME-infiltrated cells. The lollipop diagram was used to visualize the results. All *p* values were <0.05. All these procedures were performed by the R ggplot2 package. Next, the differences in immune cell scores between the low-IRLncSig-score and high-IRLncSig-score groups were calculated by Wilcoxon signed-rank analysis. The gpubr R package was used to perform this part.

To explore the correlation between immune checkpoint gene expression [including PD-1, CTLA4, hepatitis A virus cellular receptor 2 (HAVCR2), lymphocyte activation gene 3 protein (LAG3)] and IRLncSig, the Wilcoxon signed-rank test was used to evaluate the gene expression differences between the high-IRLncSig-score and low-IRLncSig-score groups. The same method was also used to explore the correlation between MGMT gene expression and IRLncSig. The ggstatsplot package was used to visualize the results.

### Enrichment analysis

The hclust function was used to identify outlier samples by sample clustering for all samples. The soft threshold of the gene expression matrix of the remaining samples was extracted by weighted correlation network analysis (WGCNA) (31). A scale-free network was constructed to verify the soft threshold. The gene expression matrix was then converted into an adjacency matrix, where the soft threshold (power value) strengthened strong connections and impaired weak correlations between genes in the adjacency matrix. Next, the adjacency matrix was converted into a topological overlap matrix (TOM) to describe the correlation between genes and used the flashclust function to perform a preliminary clustering analysis of the module on the TOM. The DynamicTreeCut algorithm was applied to identify the gene modules with more than 30 genes and merge the modules with similarities greater than 0.75. An adjusted clinical informatic matrix was extracted, and the correlation coefficient between the merged gene modules and the clinical informatic matrix was calculated. Gene modules with the highest correlation coefficients with IRLncSig scores were identified and the genes extracted in the gene modules. The Gene Ontology (GO) and Kyoto Encyclopedia of Genes and Genomes (KEGG) enrichment analyses were performed on the obtained genes to explore the biological functions of lncRNAs in IRLncSig. All of the above procedures were performed by the R packages WGCNA, ggplot2, and clusterProfiler.

## Results

### Identification of differentially expressed ir-lncRNAs

**Figure 1:**
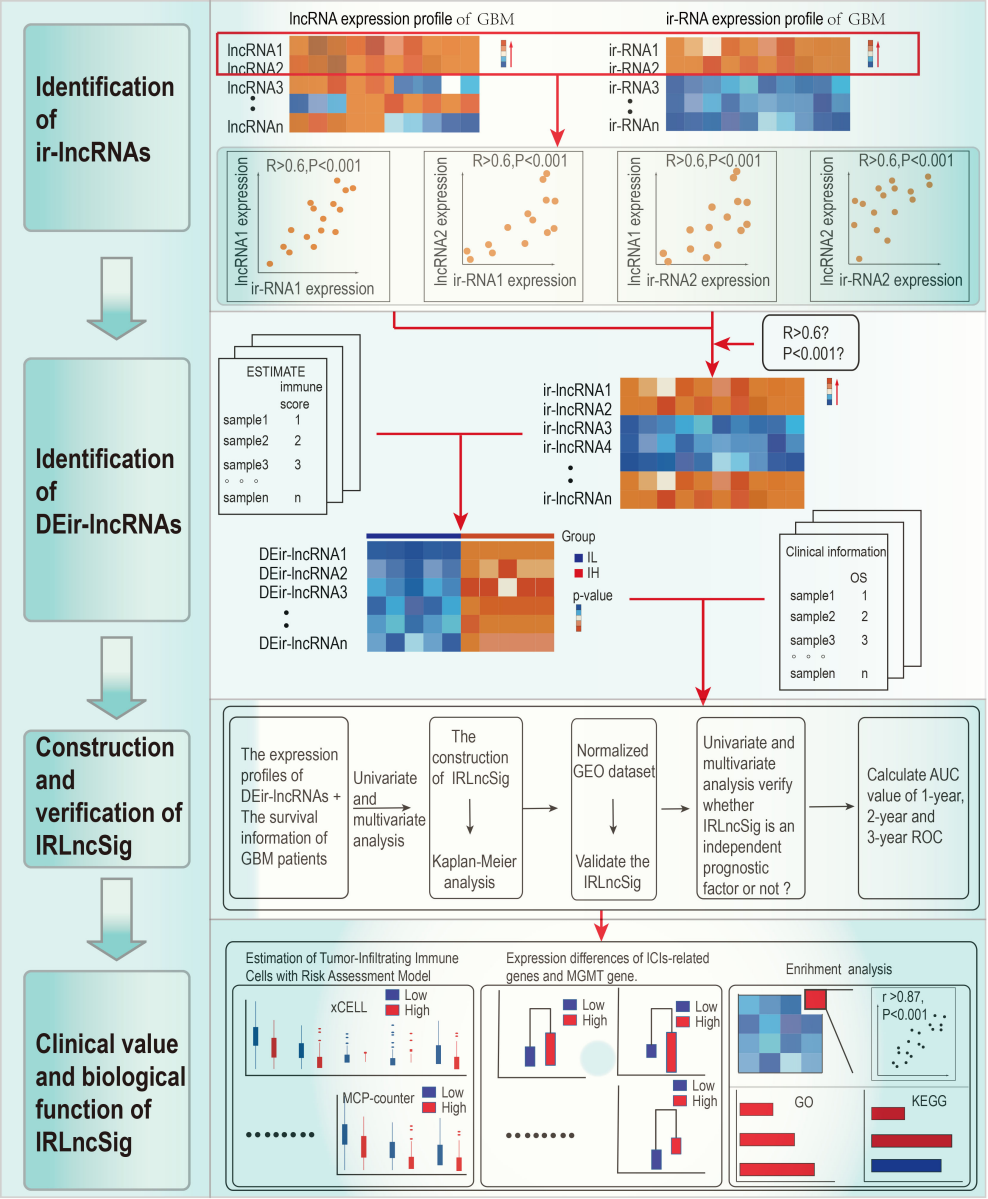
Study flow chart.

**Figure 1** shows the process flow of the study. The transcriptome profiles of the GBM project were downloaded from TCGA database. By screening pathological information and clinical information for all samples, a total of 152 samples were included in the study. All of them were evenly divided into the IL group and IH group. Next, the GTF files were used to annotate the transcriptome data, and coexpression analyses were performed between ir-genes and lncRNAs. All 787 ir-lncRNAs were identified (**Supplementary Table S1**), and 46 ir-lncRNAs were recognized as DEir-lncRNAs by differential analysis (**Supplementary Table S2**). **Supplementary Figure S1** depicts how ir-IncRNA influences gene transcription in GBM. The DEir-lncRNA expression data were visualized by a volcano plot (**Supplementary Figure S2**).

### The establishment and evaluation of IRLncSig

**Figure 2:**
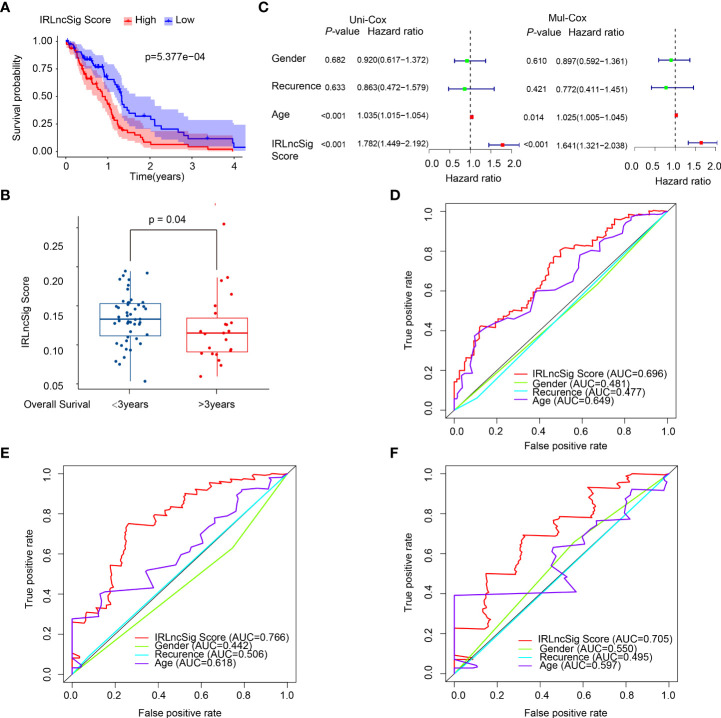
Construction and evaluation of the prognostic model. **(A)** The prognostic analysis of patients in the low-IRLncSig-score and high-IRLncSig-score groups by the Kaplan–Meier test of TCGA patients. **(B)** The Wilcoxon test of IRLncSig scores between the long-term survival group (overall survival >36 months) and short-term survival group (overall survival ≤36 months) of GEO patients. **(C)** The univariate and multivariate Cox regression prognostic analysis. **(D)** Time-dependent ROC curve analysis of IRLncSig and the other three features at 1 year. **(E)** Time-dependent ROC curve analysis of IRLncSig and the other three features at 2 years. **(F)** Time-dependent ROC curve analysis of IRLncSig and the other three features at 3 years. IRLncSig, immune-related lncRNA signature; TCGA, The Cancer Genome Atlas; GEO, Gene Expression Omnibus database; time-dependent ROC, time-dependent receiver operating characteristic.

By univariate and multivariate Cox proportional hazard regression analyses between gene expression data and prognostic information, five ir-lncRNAs were finally screened out as prognostic-related ir-lncRNAs (**Table 1**; **Supplementary Table S3**). The five prognosis-related ir-lncRNAs were used to construct IRLncSig, and the median IRLncSig score (score: 0.975) was regarded as the cutoff to distinguish between the high-IRLncSig-score group and the low-IRLncSig-score group in the cohort. Seventy-six cases were classified into the high-IRLncSig-score group, and others were classified into the low-IRLncSig-score group. The Kaplan–Meier analysis showed that patients in the low-IRLncSig-score group exhibited a longer survival time than patients in the high-IRLncSig-score group (*p* < 0.001) (**Figure 2A**). The IRLncSig score and survival status of each patient are shown in **Supplementary Figure S3A**, which suggests that patients in the low-risk group always have better clinical outcomes than those in the high-risk group. **Supplementary Figure S3B** shows that all the enrolled lncRNAs in the signature were expressed much more in the high-IRLncSig-score group than in the low-IRLncSig-score group. The effectiveness of IRLncSig was then validated by a GEO dataset. The result showed that the IRLncSig scores of the long-term survival group (overall survival >36 months) were significantly lower than the short-term survival group (overall survival ≤36 months) (**Figure 2B**). Moreover, further analysis showed that IRLncSig score and age could be independent prognostic predictors for GBM patients (**Figure 2C**, univariate Cox regression analysis: age (*p* < 0.001, HR = 1.035, 95% CI [1.015–1.054]), IRLncSig score (*p* < 0.001, HR = 1.782, 95% CI [1.449–2.192]), multivariate Cox regression analysis: age (*p* < 0.05, HR = 1.025, 95% CI [1.005–1.045]), IRLncSig score (*p* < 0.001, HR = 1.641, 95% CI [1.321–2.038])). The area under the curve (AUC) of the time-dependent ROC curve analysis of the IRLncSig score was 0.696 (1 year), 0.766 (2 years), and 0.705 (3 years) (**Figures 2D–F**).

**Table 1:** Multivariate Cox regression analyses of the five immune-related lncRNAs associated with overall survival in GBM.

Gene Symbol	Coefficient	HR	95% CI	*p*-value
AGAP2-AS1	0.230	1.259	1.100–1.441	0.001
CYP1B1-AS1	0.493	1.637	0.989–2.709	0.055
UBXN10-AS1	0.118	1.125	0.979–1.293	0.096
LINC01127	0.523	1.687	1.179–2.413	0.004
RP11-84D1.2	1.251	3.495	1.099–11.120	0.034

HR, hazard ratio; CI, confidence interval; lncRNAs, long noncoding RNAs; GBM, glioblastoma multiforme.

### Estimation of tumor-infiltrating immune cells with the risk assessment model IRLncSig

**Figure 3:**
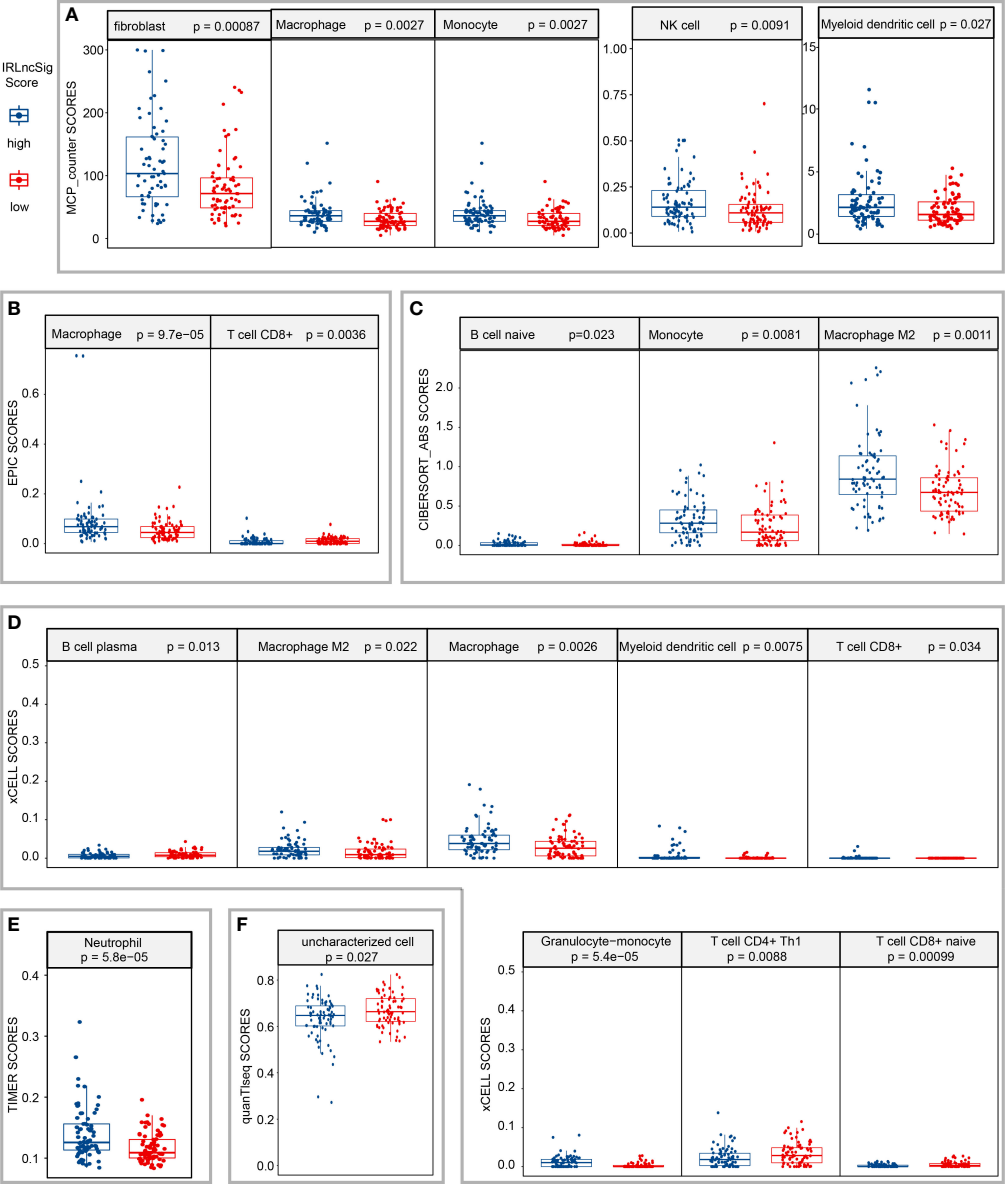
Estimation of the correlation between tumor-infiltrating cells and the risk assessment model. Wilcoxon signed-rank analysis to calculate the infiltration difference of immune cells between low- and high-IRLncSig-scores in different algorithms [**(A)** MCP-counter; **(B)** EPIC; **(C)** CIBERSORT-ABS; **(D)** xCELL; **(E)** TIMER; **(F)** quanTlseq]. IRLncSig, immune-related lncRNA signature.

LncRNAs are considered to be closely related to the tumor immune microenvironment (10, 11); thus, the correlation between IRLncSig and various infiltrating immune cells was explored in GBM. The results of Spearman’s correlation analysis demonstrated that the infiltration score of macrophages positively increased with IRLncSig scores by different algorithms, and the *p*-values of all the results were less than 0.05 (**Supplementary Figure S4**). Further Wilcoxon signed-rank analysis results showed that all subtypes of macrophages aggregated significantly more in samples with high IRLncSig scores. For other immune cells, significant differences in aggregation between the high-IRLncSig-score and low-IRLncSig-score groups were not always observed, including cancer-associated fibroblasts, monocytes, and T cells (**Figures 3A–F**).

### Expression differences of ICI-related genes and the *MGMT* gene

**Figure 4:**
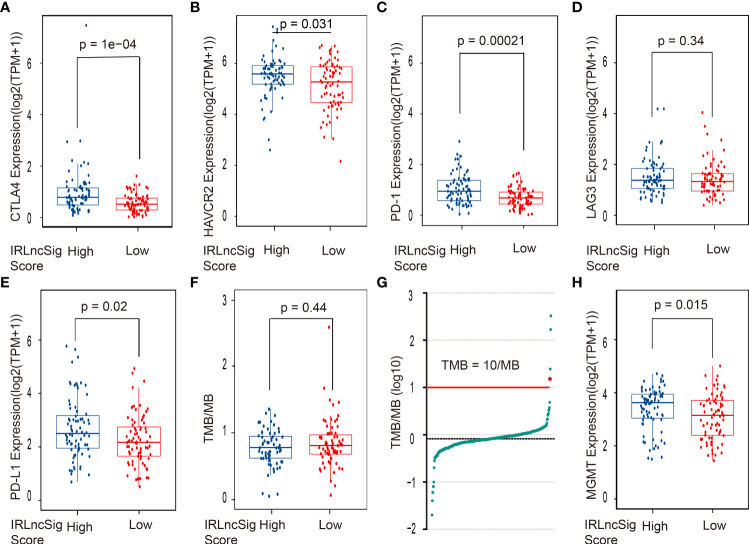
The correlation between the ICI biomarkers, the *MGMT* gene, TMB, and the risk assessment model. The high-IRLncSig-score group had significantly higher expression of **(A)** CTLA4, **(B)** HAVCR2, and **(C)** PD-1 but not **(D)** LAG3. **(E)** PD-L1 expression was higher in the High-IRLncSig-score group than in the low-IRLncSig-score group. **(F, G)** TMB does not have significant differences between the two groups and is lower than 10/MB in most cases. **(H)** The low-IRLncSig-score group was associated with lower expression of the MGMT gene. ICI biomarkers, immune checkpoint inhibitor biomarkers; MGMT, O6-methylguanine-DNA methyltransferase; TMB, tumor mutation burden; IRLncSig, immune-related lncRNA signature; CTLA4, cytotoxic T-lymphocyte-associated antigen-4; HAVCR2, hepatitis A virus cellular receptor 2; PD-1, antiprogrammed cell death protein-1; LAG3, lymphocyte activation gene 3 protein; PD-L1, programmed death ligand-1.

ICIs have been widely used in the treatment of various cancers and have shown good clinical prognostic effects (4, 5). Some clinical trials are further verifying its therapeutic effects in glioblastoma (32). Thus, the correlation between the expression of ICI-related genes and the IRLncSig score was further detected. The results showed that the expression of ICI genes in the high-IRLncSig-score group was significantly higher than that in the low-IRLncSig-score group [CTLA4 (*p* < 0.001, **Figure 4A**), HAVCR2 (*p* < 0.05, **Figure 4B**), and PD1 (*p* < 0.001, **Figure 4C**)], but no such result was observed in LAG3 (*p* > 0.05, **Figure 4D**). It has been reported that the therapeutic effects of PD-1 inhibitors are related to the expression of the programmed cell death-ligand 1 (PD-L1) gene and the tumor mutation burden (TMB). The differences in the expression of the PD-L1 gene were analyzed in different IRLncSig score groups. The results demonstrated that the expression of PD-L1 in the high-IRLncSig-score group was significantly higher than that in the low-IRLncSig-score group (*p* < 0.05, **Figure 4E**), and the TMB level of all enrolled samples did not show significant differences in different groups (*p >*0.05, **Figure 4F**), and only a few were greater than 10/MB. Moreover, the expression of the MGMT gene in the high- and low-IRLncSig-score groups was analyzed. The results demonstrated that the high-IRLncSig-score group had higher expression of the MGMT gene than the low-IRLncSig-score group (*p* < 0.05, **Figure 4H**).

### Enrichment analysis

**Figure 5:**
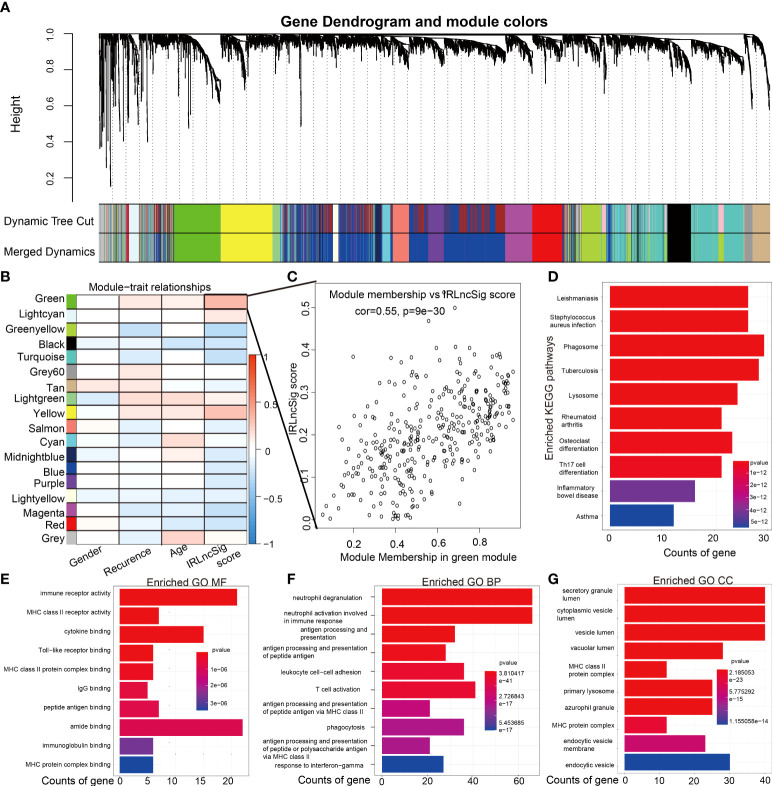
Identification of modules associated with the IRLncSig score and play enrichment analysis of ir-lncRNAs enrolled in IRLncSig. **(A)** The dendrogram of all genes is clustered based on the WGCNA algorithm. The color shows different clustering modules. **(B)** Heatmap of the correlation between the module eigengenes and clinical and molecular traits of GBM. **(C)** Correlation analysis of module membership in the selected module and IRLncSig score. **(D)** KEGG enrichment analysis is based on the genes in the selected module. **(E–G)** GO enrichment analysis is based on the genes in the selected module (molecular function (MF, Figure 6E), biological process (BP, Figure 6F), and cellular component (CC, Figure 6G)). IRLncSig, immune-related lncRNA signature; ir-lncRNAs, immune-related long noncoding RNAs; GBM, glioblastoma multiforme; KEGG, Kyoto Encyclopedia of Genes and Genomes, GO, Gene Ontology.

The hclust function was used to identify outlier samples through sample clustering and removed three outlier samples. All samples were used to develop a coexpression matrix and modules by the WGCNA algorithm. The coexpression matrix was converted into an adjacency matrix by a scale-free topology with *R* ^2^ = 0.81. By calculation, all patients were divided into 20 different modules, each labeled with a special color. Modules with similarities greater than 0.75 were selected and merged. The two gene modules were merged, and the remaining gene modules were 18 (**Figure 5A**). Next, Pearson’s correlation analysis was used to explore the association of the remaining gene modules with the IRLncSig score and other clinical features. The gene module with the highest correlation coefficients was selected for further analysis (**Figure 5B**). **Figure 5C** shows that module membership in the selected module has a close correlation with the IRLncSig score. All genes included in the selected gene module were used to perform KEGG and GO enrichment analyses to show the biological effects of lncRNAs in IRLncSig. The results of the KEGG pathway analysis revealed that significantly enriched pathways were closely related to immune cells, including phagosome and Th17-cell differentiation (**Figure 5D**). GO analysis revealed that biological effects were significantly associated with immune processes, including immune receptor activity, MHC class II receptor activity, MHC class II protein complex binding, and IgG binding [molecular function (MF, **Figure 5E**), biological process (BP, **Figure 5F**), and cellular component (CC, **Figure 5G**)]. The results indicate that lncRNAs enrolled in IRLncSig are involved in the immunological process of the TME.

## Discussion

Glioblastoma is one of the deadliest malignant tumors in humans. In the last few decades, many studies have been devoted to the treatment of GBM, but the effect is limited. The alteration of immune cells in the tumor immune microenvironment is widely recognized as one of the typical properties of tumors and has been gradually confirmed in the study of various tumors (33). Emerging studies have shown that lncRNAs play an important role in the development of the GBM TME and can partly predict the prognosis of patients (12, 13). Thus, we first attempted to construct IRLncSig by ir-lncRNAs to predict the prognosis of GBM patients and the effectiveness of ICI treatment by stratifying patients.

In this article, coexpression analyses of immune-related genes and lncRNAs were performed to select ir-lncRNAs. To efficiently select the ir-lncRNAs that have a close association with patient prognosis, the ESTIMATE algorithm was used to obtain immune scores of samples and divide them into two groups with the median. This method can help us identify ir-lncRNAs that play a dominant role in regulating the infiltration of immune cells in the TME. These DEir-lncRNAs screened by differential expression analysis in different groups were used to calculate regression analysis to obtain ir-lncRNAs related to prognosis, which was used to establish the prognostic signature. After evaluation, the results demonstrated that the prognostic model can be used as an independent prognostic risk assessment factor. Through the time-dependent ROC curve, the result further proved that the model has a certain predictive value for prognosis.

Tumor-associated macrophages (TAMs) account for 30%–40% of GBM immune infiltrating cells (34, 35) and have been shown to engage in reciprocal interactions with tumor cells to promote tumor growth and progression (36–39). Wei et al. indicated that osteopontin (OPN) is a potent chemokine for macrophages, and its blockade significantly increased the median survival time of mice by 68% (*p* < 0.05), which could be a potential therapeutic target (40). The results of the study demonstrate that TAMs are positively correlated with the increase of IRLncSig score and with statistical significance in different algorithms. When conducting intrasample comparisons between cell types, the aggregation of TAMs in the high-IRLncSig-score group was significantly higher than that in the low-IRLncSig-score group. The results of intersample comparisons of the different cell types showed that the infiltration of TAMs in most samples was significantly higher than that of other infiltrating immune cells (**Supplementary Figure S5**). As described above, in the high-IRLncSig-score group, the higher aggregation of TAMs may accelerate tumor progression and lead to a poor prognosis for GBM patients. IRLncSig can effectively distinguish the level of TAM infiltration in the TME of GBM, which means that IRLncSig may have potential clinical value in the anti-TAM treatment of GBM.

ICIs show good performance in the treatment of several tumors (41–43). In the analysis of ICI gene expression in different risk groups, the results demonstrated that the expression of most immune checkpoints (CTLA4, HAVCR2, PD-1) in the high-IRLncSig-score group was significantly higher than that in the other groups, except LAG3. Previous literature has shown that PD-1 (44) and CTLA-4 (45) exert immunosuppressive effects by weakening the immune function of T cells, and HAVCR2 suppresses IFN-g-producing T cells’, FoxP3+ Treg cells’, and innate immune cells’ (macrophages and dendritic cells) immune reactions (46). These results are consistent with the poor prognosis of patients in the high-IRLncSig-score group. Some studies have indicated that the expression of PD-L1 within the tumor microenvironment has predictive value for the response to PD-1 inhibitors in melanoma (47–49), NSCLC (50, 51), and bladder cancer (52). In the research, the expression of PD-L1 in the high-IRLncSig-score group was significantly higher than that in the low-IRLncSig-score group, which seems to indicate that the use of anti-PD-1 drugs in the high-IRLncSig-score group may achieve better efficacy. However, most existing clinical trials have shown that the therapeutic effect of PD-1 on GBM is frustrating (7, 8, 33). Further analysis indicated that the TMB [an independent predictor of PD-1 inhibitor (53)] of most included samples <10 mutations/megabase did not differ significantly between groups. Previous study indicated that the TMB for all solid tumors equal to or greater than 10 mutations/megabase may benefit from PD-1 inhibitors (54). This seems to partly explain why the performance of PD-1/PD-L1 inhibitors is poor in clinical trials of GBM. The IRLncSig may stratify patients to obtain more effective personalized ICI treatment.

Temozolomide (TMZ) is the most widely used alkylating agent in glioblastoma and is cytotoxic to cells by inducing DNA damage (55). Several studies have found that temozolomide can improve the prognosis of both primary and recurrent MGMT-methylated GBM but not the effect of unmethylated GBM (56–61). An emerging study indicates that TMZ for patients with unmethylated MGMT promoters likely has a real but marginal benefit (62). Therefore, a standard evaluation of MGMT gene expression is essential for patients with unmethylated MGMT promoters. To further expand the clinical value of IRLncSig, MGMT gene expression at different risk levels was also analyzed. The results showed that MGMT gene expression in the high-IRLncSig-score group was significantly higher than that in the other groups. This result suggests that TMZ may obtain better effectiveness in the low-IRLncSig-score group than in the high-IRLncSig-score group.

In this article, the IRLncSig constructed by five ir-lncRNAs showed a good performance in stratifying GBM patients, which may contribute to the personalized treatment of GBM patients in the future. At the same time, emerging articles have shown the potential of lncRNAs in the diagnosis and treatment of some kinds of cancers, including lung cancer, gastric cancer, colon cancer, and so on (63–66), thus the IRLncSig may contribute to developing new drugs or diagnostic tests for GBM patients hopefully.

With the improvement of technologies based on the CRISPR-Cas system, complex genetic manipulations in human cells have been made possible to treat incurable diseases (67). Meanwhile, the emergence of new drug delivery systems (including nanoparticles, Gliadel wafers, cellular carriers, etc.) further enabled the delivery of gene editing tools into the brain through the blood–brain barrier (68). Based on the personalized treatment targets found by this study, the CRISPR-Cas technology may be applied to influence gene transcription and hopefully improve the prognosis of GBM patients in the future.

Regarding the biological functions of the five lncRNAs included in IRLncSig, the IRLncSig score was used as an independent phenotype to perform correlation analysis with gene modules obtained by scale-free clustering and DynamicTreeCut algorithms to obtain the most associated gene module. The enrichment analysis of the selected gene module showed that the main biological functions were immune receptor activity, MHC class II receptor activity, MHC class II protein complex binding, and IgG binding, which verified that the five included lncRNAs are related to tumor immunity.

In this article, the IRLncSig constructed by five prognosis-related ir-lncRNAs was carefully evaluated and verified for its correlation with prognostic outcomes to ensure its clinical predictive value. At the same time, the association between IRLncSig and immune cell infiltration, ICI biomarkers, and MGMT gene expression was also analyzed. Thus, we assumed that the novel IRLncSig shows a promising clinical value in predicting the prognosis and immune landscape of GBM.

The study also has some limits. The current literature has limited reports on the ir-lncRNAs involved in constructing the IRLncSig in the research. Because the methodology applied in this study is mainly mathematical means and not molecular biological experiments, the detailed biological functions of these involved ir-lncRNAs are still not well known, so it is necessary to carry out further experimental research on this prognosis-related ir-lncRNAs in the future.

## Conclusion

The IRLncSig established by ir-lncRNA has a significant relationship with immune cell infiltration, ICI biomarkers, and MGMT gene expression, which shows a promising clinical value in predicting the prognosis and immune landscape of GBM.

## Data availability statement

The datasets presented in this study can be found in online repositories. The names of the repository/repositories and accession number(s) can be found in the article/**Supplementary Material**.

## Author contributions

Data extraction and data analysis: DS, WZ, DL, SH, YY, HY, YX, and HX. Original draft: DS, WZ, YZ, LA, and HY. Article review and editing: DS, WZ, XS, JZ, and HX. All authors contributed to the article and approved the submitted version.

## Funding

The research was funded by Chongqing Natural Science Foundation (Application number: 2022NSCQ-MSX0963).

## Conflict of interest

The authors declare that the research was conducted in the absence of any commercial or financial relationships that could be construed as a potential conflict of interest.

## Publisher’s note

All claims expressed in this article are solely those of the authors and do not necessarily represent those of their affiliated organizations, or those of the publisher, the editors and the reviewers. Any product that may be evaluated in this article, or claim that may be made by its manufacturer, is not guaranteed or endorsed by the publisher.
