# Supplementary material for: Computational identification of immune-related lncRNA signature for predicting the prognosis and immune landscape of human glioblastoma multiforme
Source: Front Immunol. 2022 Aug 12;13:932938. doi: 10.3389/fimmu.2022.932938 (PMC9412749; doi:10.3389/fimmu.2022.932938)
Supplement: Supplementary file 1 [file DataSheet_1.docx]

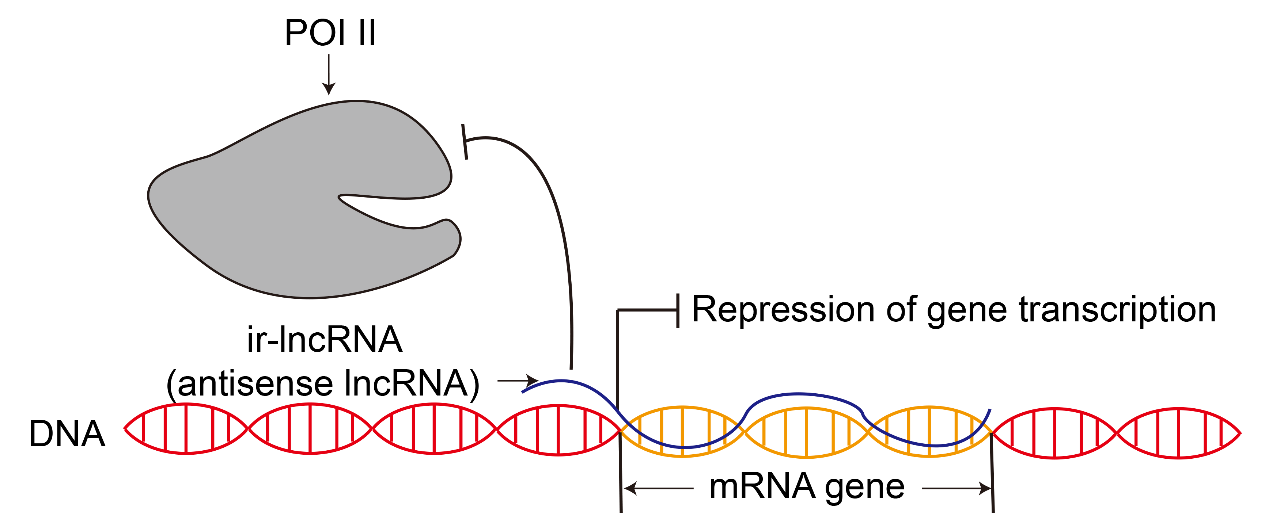


Supplementary Figure 1. The mechanism of how ir-IncRNA influences gene transcription in GBM. The ir-lncRNA (antisense lncRNA) interfere with gene transcription by preventing Pol II to combine with mRNA gene.

RNA: Ribonucleic Acid, mRNA: messenger RNA, ir-IncRNA: immune-related long noncoding RNAs, GBM: Glioblastoma Multiforme, Pol II: RNA polymerase II.

References:

1. Pelechano V, Steinmetz LM. Gene regulation by antisense transcription. Nat Rev Genet(2013)14(12):880-93. doi: 10.1038/nrg3594

2. Quinn JJ, Chang HY. Unique features of long non-coding RNA biogenesis and function. Nat Rev Genet (2016)17(1):47-62. doi: 10.1038/nrg.2015.10


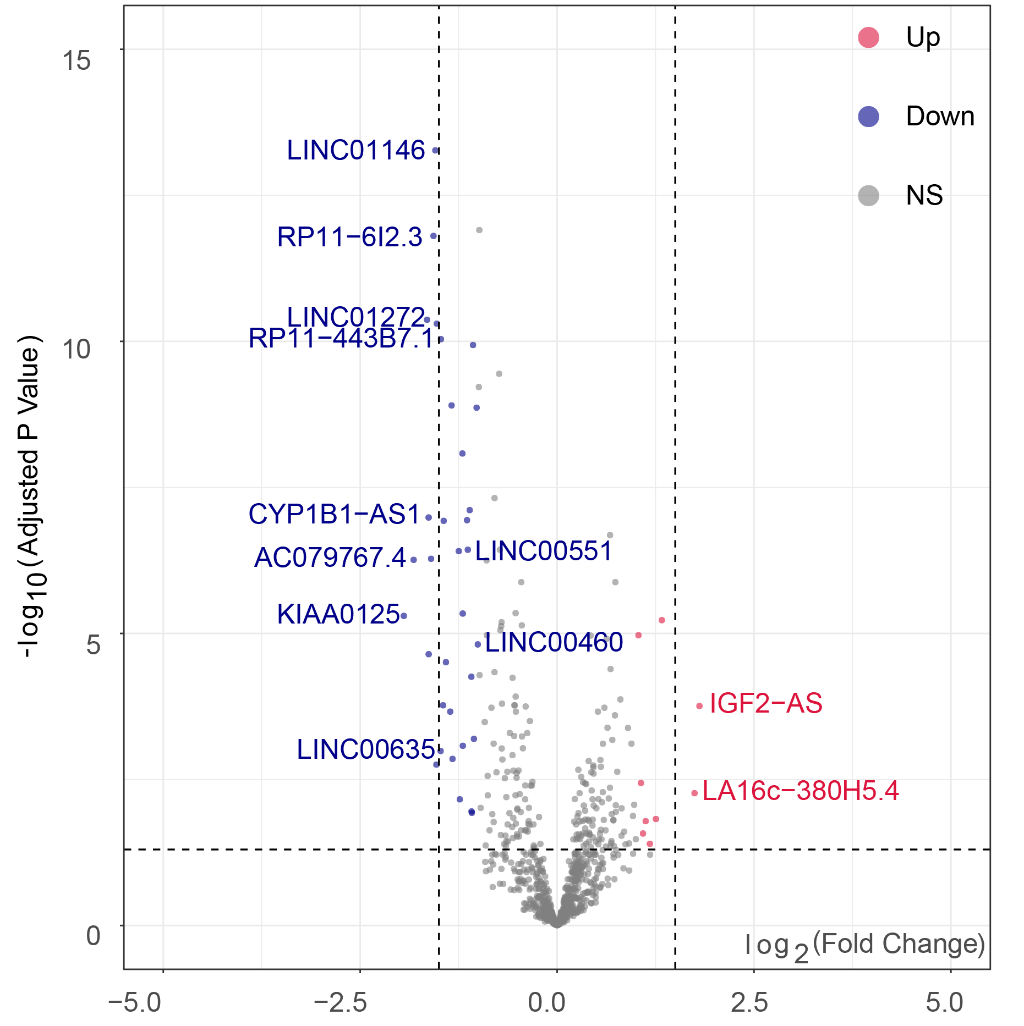


Supplementary Figure 2. The volcano plot of DEir-lncRNA expression.

DEir-lncRNA：differentially expressed immune-related long noncoding RNAs


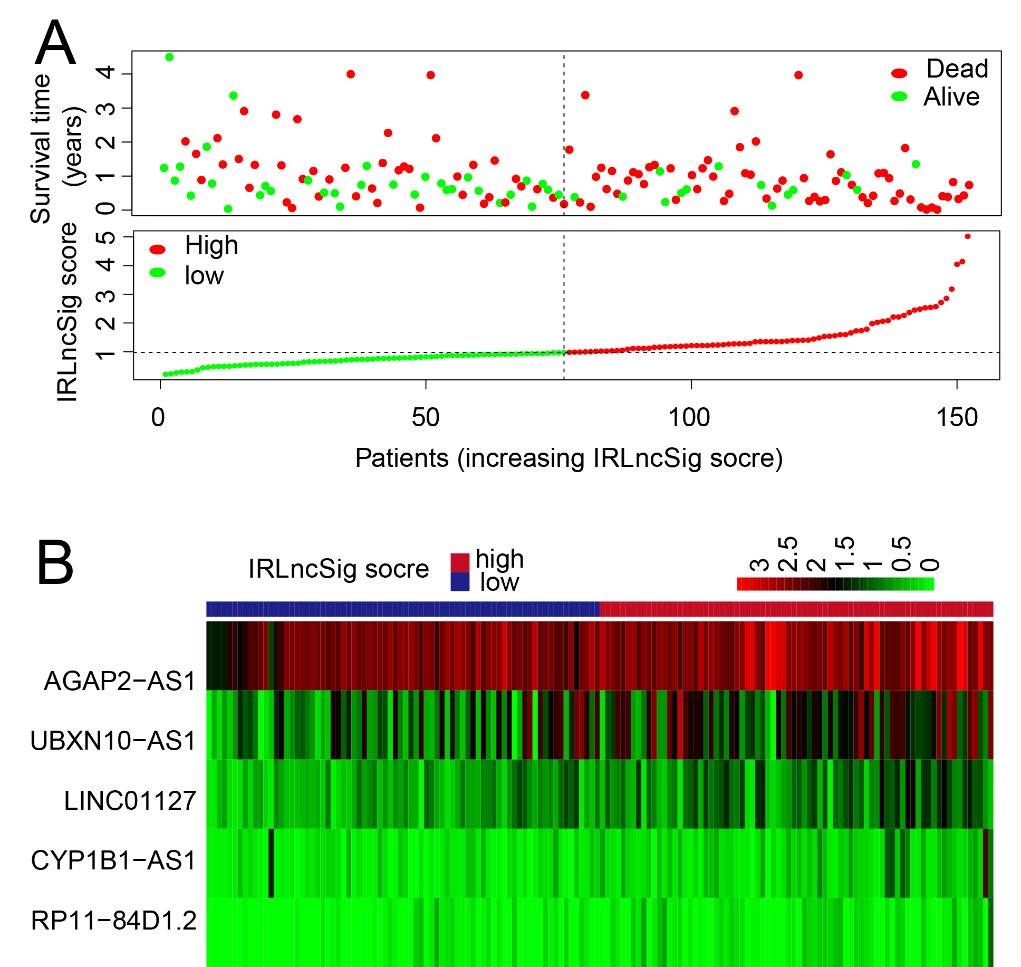


Supplementary Figure 3. (A) Survival outcome with the increasing IRLncSig score of each TCGA case. (B) The enrolled LncRNAs expression patterns with increasing IRLncSig score.

IRLncSig: immune-related lncRNA signature, TCGA: The Cancer Genome Atlas, LncRNAs: long noncoding RNAs


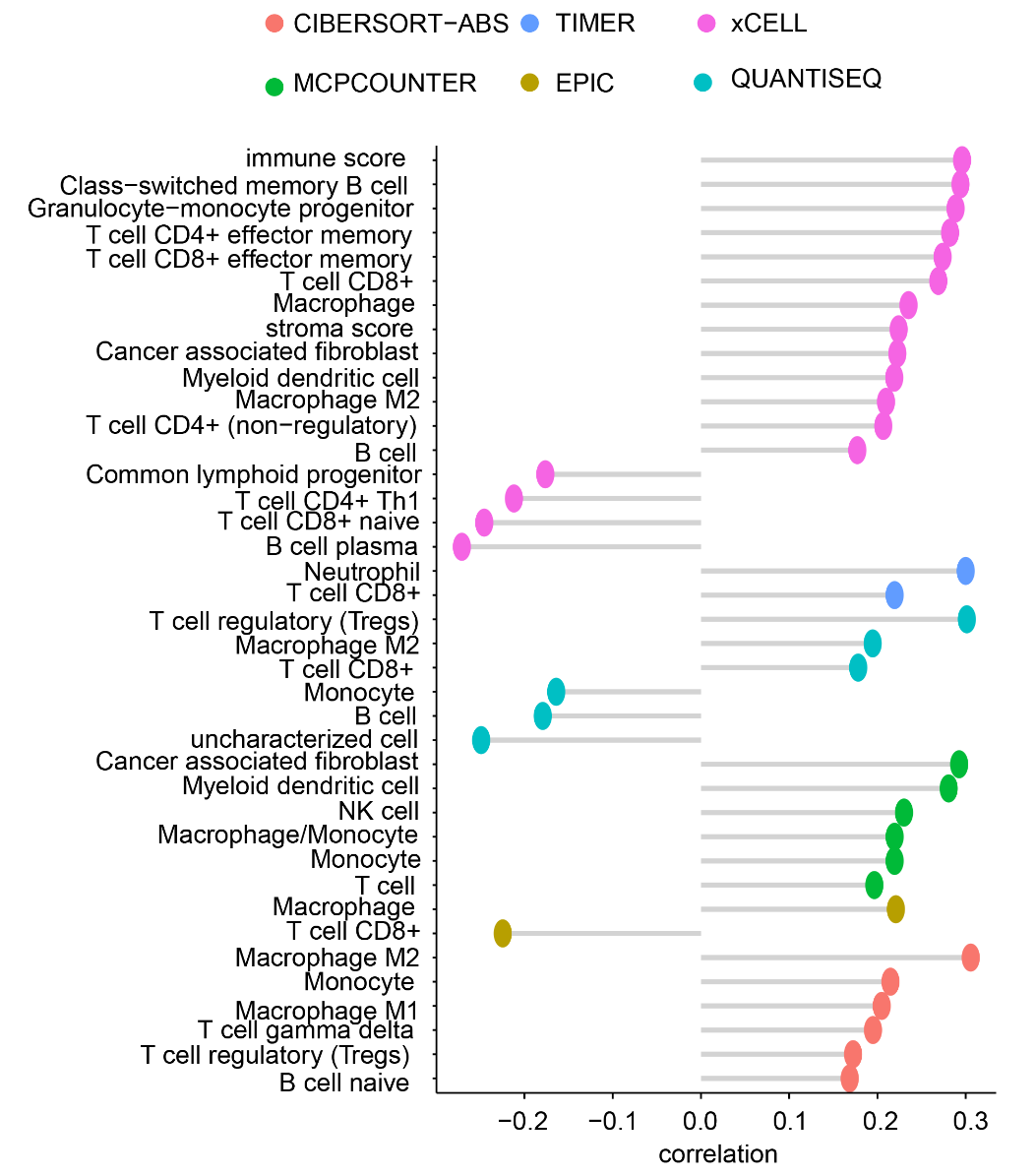


Supplementary Figure 4. The Spearman correlation analysis was used to calculated the association between IRLncSig score and tumor-infiltrating immune cells (all *p*-value < 0.05).

IRLncSig: immune-related lncRNA signature


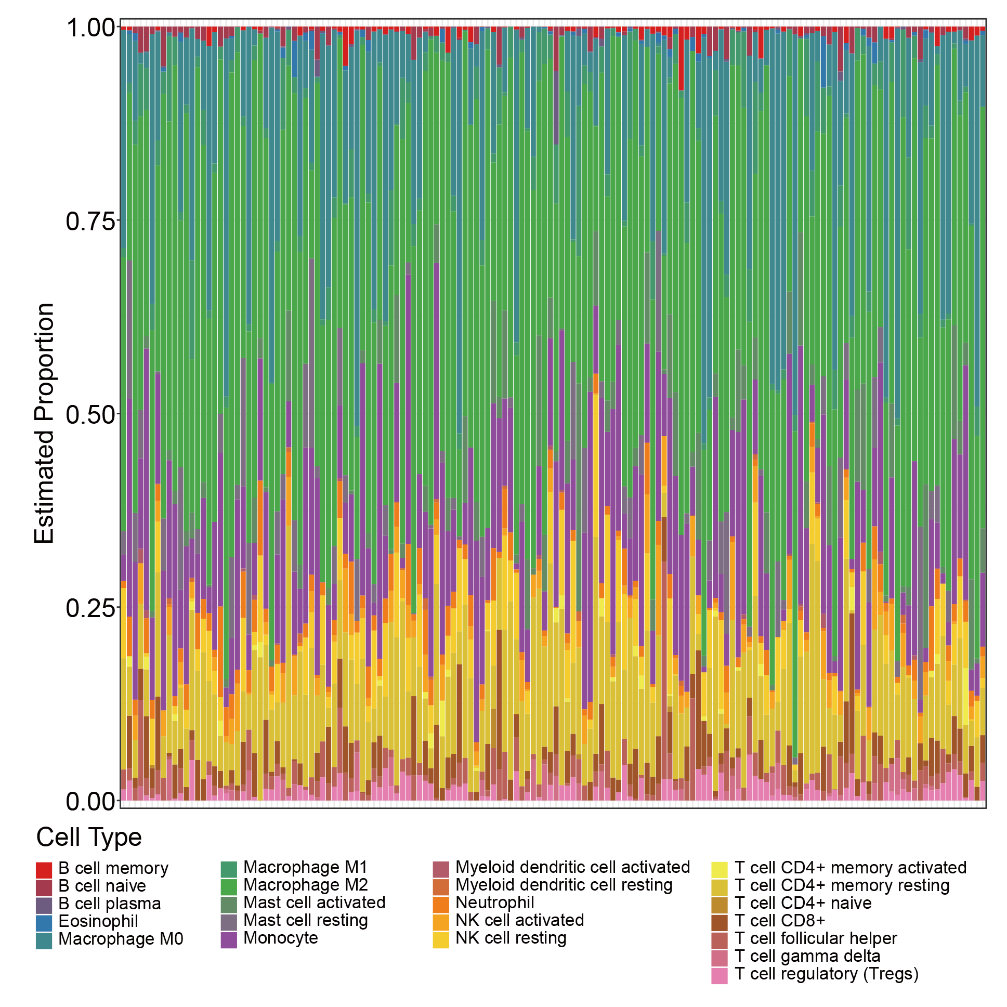


Supplementary Figure 5. Intersample comparisons of the different cell type. The infiltration of TAMs in most samples was significantly higher than that of other immune infiltrating cells.

TAMs: Tumor-associated macrophages

Supplementary Table 3: Information about five shortlisted lncRNAs

| Gene symbol | Genomic Locations | Functional Annotation |
| --- | --- | --- |
| AGAP2-AS1 | chr12:57,726,239-57,728,356 | positive regulation of RNA polymerase II transcriptional preinitiation complex assembly; regulation of RNA polymerase II transcriptional preinitiation complex assembly; |
| CYP1B1-AS1 | chr2:38073447-38232317 | regulation of postsynaptic membrane neurotransmitter receptor levels;  protein palmitoleylation; |
| UBXN10-AS1 | chr1:20184300- 20186658 | negative regulation of T cell mediated immunity;  regulation of inflammatory response to antigenic stimulus;  negative regulation of lymphocyte mediated immunity; |
| LINC01127 | chr2:101983464- 101985545 | Unknown |
| RP11-84D1.2 | Unknown | Unknown |

chr: chromosome.

References:

1. Hou M, Tang X, Tian F, Shi F, Liu F, Gao G. AnnoLnc: a web server for systematically annotating novel human lncRNAs. BMC Genomics (2016)17(1):931. doi: 10.1186/s12864-016-3287-9

2. Ke L, Yang DC, Wang Y, Ding Y, Gao G. AnnoLnc2: the one-stop portal to systematically annotate novel lncRNAs for human and mouse. Nucleic Acids Res(2020)48(W1): W230-W8. doi: 10.1093/nar/gkaa368
